# Seed nutritional quality in lentil (*Lens culinaris*) under different moisture regimes

**DOI:** 10.3389/fnut.2023.1141040

**Published:** 2023-06-16

**Authors:** Ruchi Bansal, Ram Swaroop Bana, Harsh K. Dikshit, Harshita Srivastava, Swati Priya, Sunil Kumar, Muraleedhar S. Aski, N. K. Prasanna Kumari, Sanjeev Gupta, Shiv Kumar

**Affiliations:** ^1^ICAR-National Bureau of Plant Genetic Resources, New Delhi, India; ^2^ICAR-Indian Agricultural Research Institute, New Delhi, India; ^3^National Institute of Science Communication and Policy Research, New Delhi, India; ^4^Indian Council of Agricultural Research, New Delhi, India; ^5^International Center for Agriculture in Dryland Areas, Rabat, Morocco

**Keywords:** biofortification, food, lentil, legume, nutrition, protein, water stress, yield

## Abstract

The world’s most challenging environmental issue is climate change. Agricultural productivity and nutritional quality are both substantially threatened by extreme and unpredicted climate events. To develop climate resilient cultivars, stress tolerance along with the grain quality needs to be prioritized. Present study was planned to assess the effect of water limitation on seed quality in lentil, a cool season legume crop. A pot experiment was carried out with 20 diverse lentil genotypes grown under normal (80% field capacity) and limited (25% field capacity) soil moisture. Seed protein, Fe, Zn, phytate, protein and yield were recorded in both the conditions. Seed yield and weight were reduced by 38.9 and 12.1%, respectively, in response to stress. Seed protein, Fe, Zn, its availability as well as antioxidant properties also reduced considerably, while genotype dependent variation was noted with respect to seed size traits. Positive correlation was observed between seed yield and antioxidant activity, seed weight and Zn content and availability in stress. Based on principal component analysis and clustering, IG129185, IC559845, IC599829, IC282863, IC361417, IG334, IC560037, P8114 and L5126 were promising genotypes for seed size, Fe and protein content, while, FLIP-96-51, P3211 and IC398019 were promising for yield, Zn and antioxidant capacity. Identified lentil genotypes can be utilized as trait donors for quality improvement in lentil breeding.

## 1. Introduction

Climate change is detrimental to all the dimensions of food and nutritional security. Climate unpredictability has disturbed the global food production, accessibility, utilization as well as food system stability (1). During the past 40 years, agricultural productivity has suffered a significant setback as a result of climatic variabilities like extreme temperatures, flooding, drought, and an increase in the occurrence of pests and diseases (2). The United Nations has set Sustainable Development Goals (SDGs) to achieve a better and sustainable future for all till 2030. The accomplishment of “Zero Hunger” and eradication of poverty is the most important goal among SDGs. To achieve these goals, agriculture and food systems must be sustainable, resource- efficient, nutrition-sensitive, and climate-smart.

Legumes play an important role in food and nutritional security and contribute roughly 10% of daily protein consumption and 5% of daily energy intake (3). They also contain considerable levels of vitamins (thiamin, riboflavin, pyridoxine, vitamin K, E, B and folic acid) and minerals (Ca, Fe, Zn, Mg, and lysine). Though the food legumes grow in a diverse range of environments, abiotic stresses such as drought, heat/temperature, salinity, and heavy metals adversely affect grain yield and quality (4, 5). Water stress is among the most critical factors limiting the production of legumes, particularly in the arid and semi-arid tropics. Water limitation during the flowering/grain filling is highly detrimental to grain yield and quality (6). High temperature/water stress may lead to early senescence, shorten seed filling duration and affect remobilization of assimilates from source to sink (7). Grain development is mostly limited by stress-induced reductions in assimilate supply (8, 9). Poor soil moisture deteriorated grain quality in wheat by affecting protein composition and dietary fibre content (10), while, grain N, P, Fe, and Zn levels along with the total grain protein in chickpea (11). In rice, grain length, width, and total milling recovery decreased, and chalkiness increased under the water deficit (12). Terminal stress altered the fatty acid composition of soybean seeds, which depreciated the oil content, quality, and stability (13). Though different studies have shown the negative impact of water stress on yield in major cereals and legumes, nutritional aspects have not gained much attention.

Lentil (*Lens culinaris* L.) is an important legume crop, which is primarily cultivated in Canada, India, and Turkey (14). Lentil seeds are highly rich in protein (20–30%), low digestible carbohydrates (20%), fat (1.0%), and vitamins (15). The high concentrations of prebiotic or low-digestible carbohydrates in lentils, such as resistant starch (75 mg g^−1^), raffinose-family oligosaccharides (40.7 mg g^−1^), sugar alcohols (14.2 mg g^−1^) and fructo-oligosaccharides (0.62 mg g^−1^) contribute to its health benefits (16). In black gram and green gram, water stress during the post-flowering growth phase may reduce up to 70% of grain yield (17). Heat individually and in combination with water stress declined grain Fe, Zn and crude protein content in lentils. Combined stress was more detrimental to lentil yield and quality compared to heat individually (18). Since lentil is a highly nutritious legume crop, there is a need to generate information on effect of water limitation on grain quality to ensure nutritional security amid climatic variability. Therefore, we hypothesized that water limitation may deteriorate the seed quality in lentils and identification of genotypes with better yield and quality under stress should be targeted in lentil improvement breeding.

Keeping this in view, the present study aimed to (i) determine the effect of limited soil moisture on nutritional quality in diverse lentil genotypes (ii) analyze the relationship among yield and quality traits in different water regimes (iii) selection of superior lentil genotypes in terms of seed yield and quality in response to stress.

## 2. Materials and methods

### 2.1. Experimental material

A set of 20 lentil accessions (P3211, IC560037, IC398019, P8110, L5126, IC278791, IC559845, IG129185, IG334, IC282863, IC201678, IC361417, IC559829, IC279627, IC201676, IC208327, EC78391, P8114, FLIP-96-51, JL3) was assessed for their yield and quality response to water stress in controlled conditions during 2019–20 and 2020–21. The seeds were obtained from National Genebank, ICAR- National Bureau of Plant Genetic Resources, New Delhi, India.

### 2.2. Experimental conditions

The experiment was conducted in a randomized control block design with four replications (10 pots per replication) and two treatments during rabi season at ICAR-National Bureau of Plant Genetic Resources, New Delhi (28.6331°N, 77.1525°E). Pots with 14″ diameter were filled with the top field soil (sandy loam, pH 7.0) and farmyard manure in a ratio of 1:1. The potting mixture was supplemented with Tricalcium phosphate fertilizer (10 mg kg^−1^) before pot filling. Plants were maintained at 80% field capacity till flowering. Field capacity was maintained gravimetrically by measuring the pots regularly and supplying only the required amount of water (19). At the onset of flowering, water stress was implemented in one set of genotypes by restricting the water supply till 25% field capacity is reached. Thereafter, stressed plants were maintained at the same field capacity till harvesting. Normal plants were maintained at 80% field capacity till harvesting. Before sowing, seeds were treated with 1% Sodium hypochlorite solution followed by thorough washing. Seed germination was carried out in dark at 22°C. After emergence, five seedlings were transferred to pots. Later on, two plants were maintained in each pot. Plants from normal and stress were harvested at maturity for recording the yield and quality parameters.

### 2.3. Grain yield and test weight

At maturity, the plants were harvested. Grain yield was measured by thrashing 10 plants from each replication. Test weight was recorded from three replications for 100 seeds per replication.

### 2.4. Estimation of seed quality traits

Seed samples from three replications were taken for quantifying Fe and Zn content from both water regimes. Samples were digested using the standard diacid digestion method. Total Fe and Zn were measured by using atomic absorption spectroscopy (20). N content was determined in seed samples using the Kjeldahl method (21). Seed protein was calculated by multiplying the N content with 6.25 as a conversion factor.

Phytic acid (PA) was analyzed in the seed samples using the Megazyme kit (22) as per standard assay procedure for P issued by phytase and alkaline phosphatase. The inositol phosphates are acid extracted, then treated with a phytase that is selective for PA (IP6), and the lower myo-inositol phosphate forms. Further reaction with alkaline phosphatase produces the final phosphate from myo-inositol phosphate (IP1) which is relatively phytase resistant. A modified colorimetric method was used to determine the total released phosphate. Inorganic phosphate was quantified as P from a calibration curve developed using standards of known P concentration. PA and Zn contents were converted to moles and the ratio was calculated accordingly. DPPH radical scavenging activity assay was carried out in a methanolic extract of lentil genotypes spectrophotometrically (23). The activity was calculated using the below- mentioned equation:

$$\begin{array}{l} \mathrm{DPPH free radical scavenging activity}\;\left(\%\right)=\\ \left(1-\mathrm{Absorbance of sample}/\mathrm{Absorbance of control}\right)\times 100 \end{array}$$

### 2.5. Seed size traits

Seed size (area, length and breadth) was studied from each replication by scanning the seeds of each genotype with a flatbed scanner (Canon LiDE 110 version 1.3.00). The scanned images were analyzed using Grain Size & Shape Properties, a MATLAB based software developed by ICAR-Central Institute of Agriculture Engineering, Bhopal, India.

### 2.6. Statistical analysis

Data was analyzed using R software. The least significant difference was calculated at 5 and 1% p level. Pearson’s correlation coefficient analysis and principal component analysis were done to study the correlation and identification of traits contributing to yield and quality under normal and stress conditions. The clustering of the genotypes was done using Ward’s method in Microsoft Excel.

## 3. Results

In both normal and stress conditions, lentil genotypes were assessed for yield (seed plant^−1^, test weight), seed size (length, width, and area), and quality parameters (Fe, Zn, protein, phytic acid, PA:Zn, DPPH radical scavenging activity). The effects of genotype (G), environment (E), and their interaction (G × E) were highly significant for all examined characteristics at *p* < 0.001, with the exception of test weight (TW) at *p* < 0.01.

### 3.1. Effect of water stress on yield traits

Seed yield plant^−1^ (SY) and test weight (TW) decreased significantly because of stress (Table 1). Effect of stress was more on yield compared seed weight as shown by reduction in mean SY (38.9%) and TW (12.1%). Coefficient of variation was 6.3% for yield and 7.34% for seed weight under normal condition, which reduced under stress environment (Table 1). G, E and G × E interaction effects were highly significant for SY at *p* < 0.001 and TW at *p* < 0.01 (Table 2).

**Table 1:** Summary statistics for seed quality, size and yield traits of lentil genotypes.

	Trt	Fe (ppm)	Zn (ppm)	PRT (%)	PA (mg g^−1^)	PA:Zn	DPPH (%)	AREA (mm^2^)	LEN (mm)	BRD (mm)	SY gm plant^−1^	TW (gm)
Mean	N S	52.79 40.02	50.63 34.90	23.26 17.37	8.50 11.25	12.35 25.08	6.81 3.75	9.69 12.94	3.79 4.51	3.22 3.79	5.17 3.16	2.57 2.26
Max	N S	84.05 52.65	66.80 49.60	25.81 22.50	13.30 13.90	18.08 32.56	8.40 5.60	29.32 18.37	6.28 6.88	6.15 4.55	6.92 4.34	3.57 3.37
Min	N S	31.30 27.30	36.60 20.40	17.84 12.66	5.10 9.70	8.05 19.20	4.20 1.40	6.37 8.90	3.15 3.68	2.58 3.13	3.13 2.17	2.21 1.47
SD	N S	12.61 6.74	8.62 8.98	2.02 2.26	1.98 1.36	0.05 0.12	1.25 0.87	4.77 2.61	0.63 0.68	0.73 0.42	0.82 0.52	0.35 0.36
CV (%)	N S	4.17 5.93	5.87 3.89	11.51 7.67	4.29 8.27	3.40 2.83	5.45 4.31	2.03 4.96	6.32 6.63	4.41 9.02	6.30 6.07	7.34 6.28

Mean, Average value; SD, standard deviation; Min, minimum value; Max, maximum value; Fe, Iron; Zn, Zinc; PRT, protein; PA, phytic acid; DPPH, DPPH scavenging activity; AREA, seed area; LEN, length; BRD, breadth; SY, seed yield; TW, test weight; Trt, treatment; N, normal; S, water stress.

**Table 2:** Analysis of variance (ANOVA) for seed size, quality and yield traits of lentil genotypes.

	Fe	Zn	PRT	PA	PA:Zn	DPPH	AREA	LEN	BRD	SY	TW
G	501.51***	344.62 ***	18.99 ***	13.96 ***	3.50 ***	5.47 ***	62.98 ***	1.51 ***	1.50 ***	2.38 ***	0.65 ***
E	219.36***	9216.92***	1039.35***	226.82 ***	9.93 ***	281.39 ***	316.14 ***	15.48 ***	9.84 ***	120.98 ***	3.05***
GxE	318.62***	239.65 ***	8.61 ***	3.34 ***	1.50 ***	1.51 ***	25.80 ***	1.09 ***	0.66 ***	0.43 ***	0.10**

*p < 0.05, **p < 0.01,***p < 0.001. Fe, Iron; Zn, Zinc; PRT, protein; PA, phytic acid; DPPH, DPPH scavenging activity; AREA, seed area; LEN, length; BRD, breadth; SY, seed yield; TW, test weight; G, genotype; E, environment.

### 3.2. Effect of water stress on grain quality

Fe content ranged 31.3–84.1 ppm with a mean value of 52.8 ppm and reduced by 24.2% on exposure to stress (Table 1). Similarly, Zn content recorded the depreciation of 31.1% compared to the control. Zn content varied from 36.6–66.8 ppm under normal and 20.4–49.6 ppm under stress conditions. Grain protein ranged from 17.8–25.8% among different genotypes and. it reduced severely by 25.3% (Table 1).

Mean PA content ranged from 5.1–13.3 in normal with a mean of 8.5 (Table 1). Stress increased PA by 32.4%. Genotypic variation was the highest for PA (11.5%) among all the recorded traits in no stress condition. Similarly, the mean PA:Zn ratio became two -fold in water stress. PA:Zn ratio ranged from 8.05–28.08 in normal conditions. DPPH free radical scavenging activity ranged 4.2–8.4 in normal and declined remarkably by 44.9% on exposure to stress (Table 1). G, E and G × E effects were significant for all the quality traits at *p* < 0.001 (Table 2).

### 3.3. Effect of water stress on seed size

Limited moisture availability resulted in a significant increase in mean seed length, breadth, and area by 18.9, 17.7 and 33.5%, respectively, (Table 1). Variation was the highest for BRD (9.02%) under drought compared to all observed traits. The range for seed LEN was 3.2–6.3 mm, BRD 2.6–6.2 mm and AREA 6.4–29.3 mm^2^ under normal condition. ANOVA showed the existence of highly signification G, E, G × E interaction effects for all the size traits (Table 2).

### 3.4. Association between yield, seed size and quality traits

**Figure 1:**
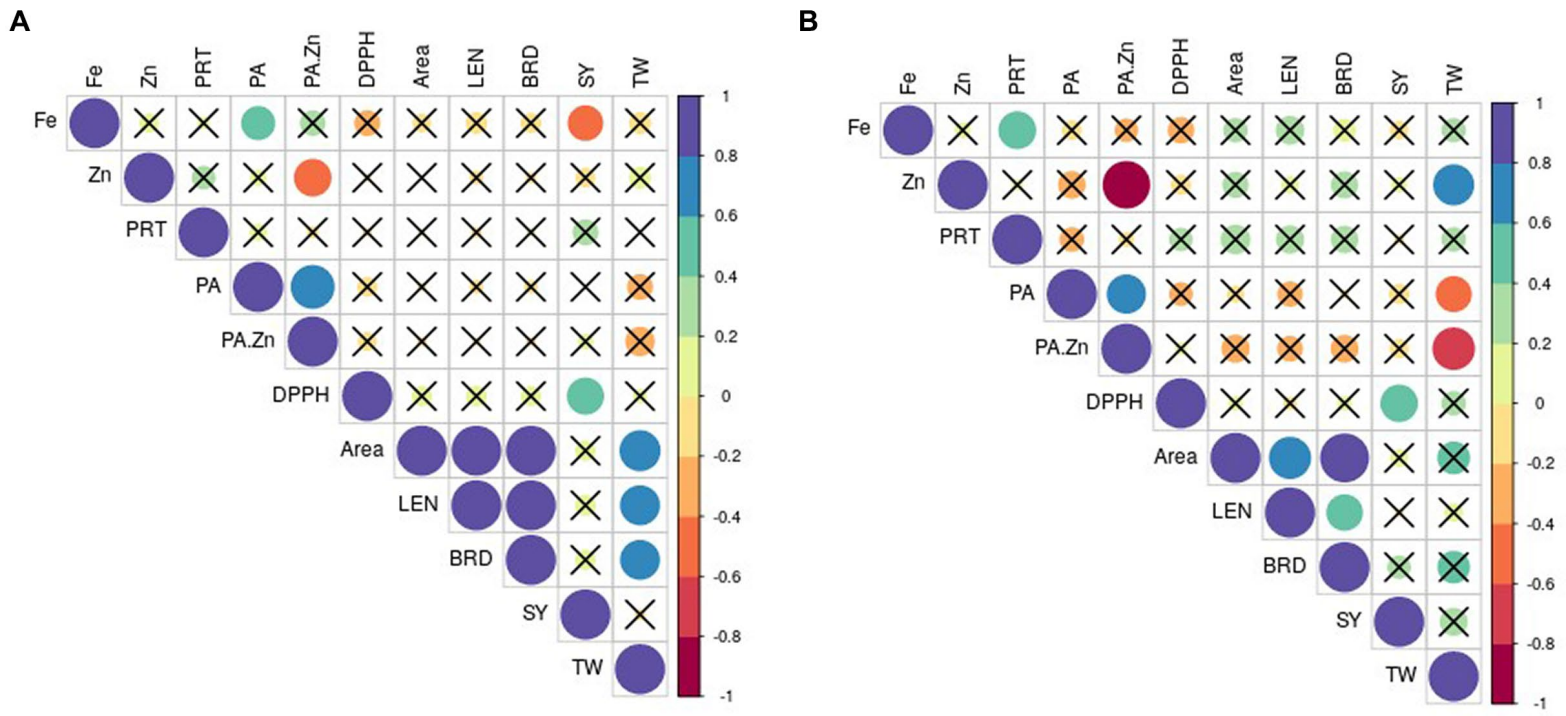
Pearson correlation’s coefficient between selected traits in **(A)** normal and **(B)** stress conditions. The non-significant correlations (*p* < 0.05) are indicated with a cross in the individual cells. PRT, protein; PA, phytic acid; DPPH, DPPH scavenging activity; LEN, length; BRD, breadth; SY, seed yield; TW, test weight.

The correlations between yield and other quality traits are depicted in Figure 1. Seed yield had negative correlation to Fe (*r* = 0.49, *p* < 0.05) and a positive correlation to DPPH activity (*r* = 0.54, *p* < 0.05) under normal condition (Figure 1A). Grain weight was positively associated with seed length (*r* = 0.63 *p* < 0.05), breadth (*r* = 0.62 *p* < 0.05) and area (*r* = 0.66 *p* < 0.05). No correlation between TW and seed nutritional quality traits existed in normal condition.

Under stress, yield showed a significant positive correlation to DPPH activity only (*r* = 0.55 *p* < 0.05; Figure 1B). Seed weight exhibited a positive association with Zn (*r* = 0.66 *p* < 0.05) and negative correlation to PA (*r* = 0.51 *p* < 0.05), PA/Zn (*r* = 0.71, *p* < 0.05) in stress conditions.

### 3.5. Principal component analysis

Principal component analysis (PCA) was used to determine the major traits accountable for genotypic variability under both the treatments. Eigenvalues, variance, and cumulative variances are shown in Table 3. We identified four principal components explaining 81.21% variability under normal and 80.26% variability under stress conditions.

**Table 3:** Extracted Eigenvalues and vectors associated with the first four principal components (PC) under normal and stress condition.

Particulars	Treatment	PC1	PC2	PC3	PC4
Eigenvalues	Normal Stress	3.67 3.85	2.09 1.97	1.82 1.72	1.35 1.29
variance (%)	Normal Stress	33.40 35.02	19.00 17.93	16.52 15.58	12.30 11.72
Cumulative variance (%)	Normal Stress	33.40 35.02	52.40 52.95	68.92 68.53	81.22 80.25
Traits		Coefficient vectors			
Zn	Normal Stress	0.19 0.21	0.03 0.32	0.52–0.25	0.76–0.45
Fe	Normal Stress	0.36 0.34	−0.17 -0.30	0.35–0.30	−0.12 0.22
Protein	Normal Stress	0.17 0.23	0.01 0.28	−0.65 -0.31	0.45–0.57
Phytic acid	Normal Stress	−0.34 -0.27	0.17 0.28	0.22 0.06	0.26 0.38
PA/Zn ratio	Normal Stress	−0.39 -0.39	0.24 0.32	−0.15 0.29	0.16–0.06
DPPH scavenging activity	Normal Stress	0.37 0.06	−0.10 -0.23	−0.19 0.62	0.14–0.24
Area	Normal Stress	−0.25 0.39	−0.51 0.33	−0.03 0.20	0.13 0.29
Length	Normal Stress	−0.28 0.30	−0.42 0.38	−0.05 0.04	0.14 0.08
Breadth	Normal Stress	−0.27 0.37	−0.48 0.28	−0.05 0.23	0.10 0.34
Seed yield	Normal Stress	0.37 0.13	−0.09 -0.29	−0.24 0.51	0.13 0.02
Test weight	Normal Stress	0.22 0.40	−0.45 -0.29	0.13–0.01	−0.16 -0.05

**Figure 2:**
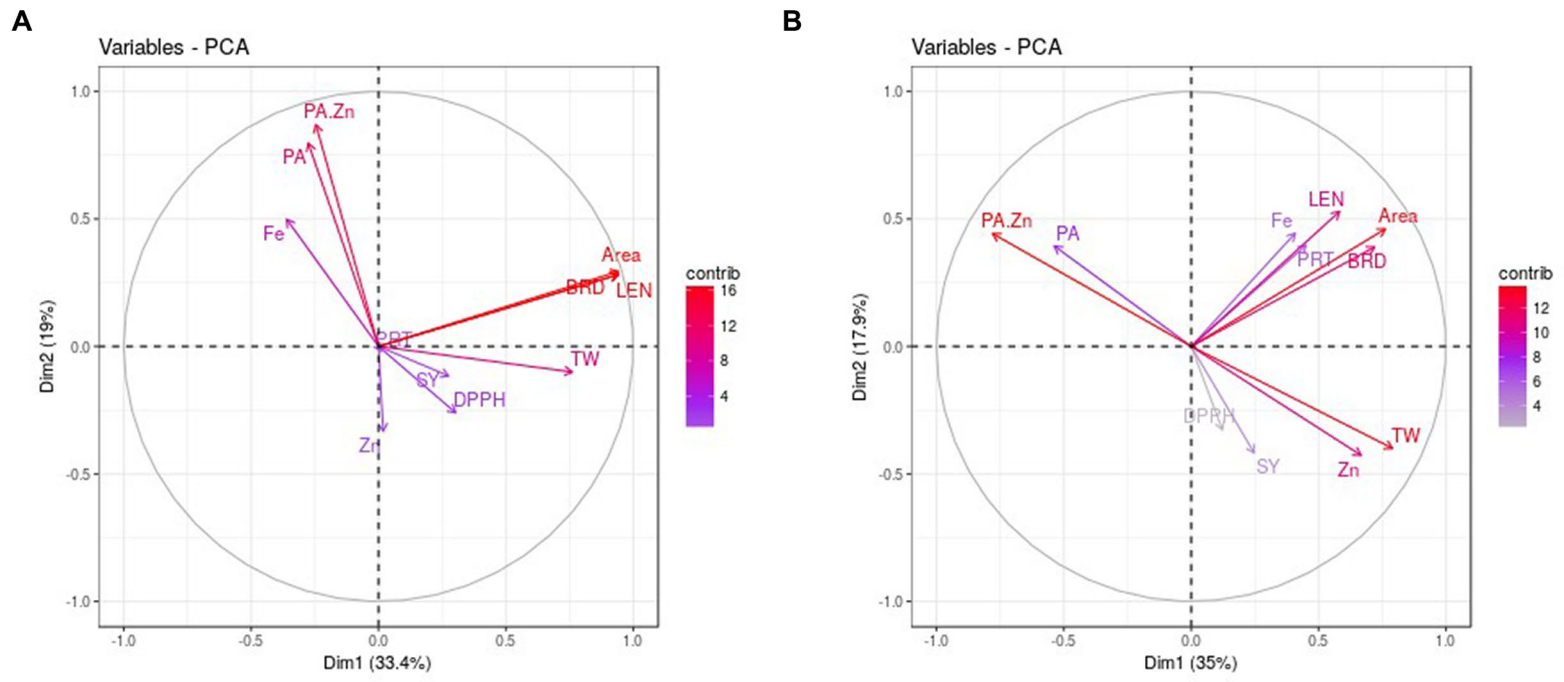
PCA biplots of studied traits in normal **(A)** and water stress **(B)** conditions. PRT: protein, PA: phytic acid, DPPH: Total antioxidant activity, LEN: length, BRD: breadth, SY: seed yield, TW: test weight.

The PCA analysis revealed that PC1 contributed 33.40% of total variability and it was positively associated with grain quality parameters while grain size traits were negatively correlated in normal conditions (Table 3). The PC2 accounted for 19% and was associated positively with Zn, protein, and phytic acid. Similarly, PC3 contributed to 16.52% of the variability and had a significant association with Zn, Fe, and PA, TW and a low association with grain yield. PC4 explained a 12.30% highly positive association with Zn, protein, and other studied traits except for Fe and grain weight (Table 3). PCA biplot analysis considering PC1, and PC2 identified three trait groups among studied genotypes (Figure 2A). Seed size, quality (Except Fe, PA and PA:Zn ratio) and yield traits were the major contributors to PC1 and were highly correlated in the genotypes present in group I, and IV. Seed Fe, phytate and PA:Zn ratio contributed to PC2 and was correlated in genotypes present in group II. Genotypes in group III were associated to yield and quality traits contributing to PC1.

In stress, PC1 explained 35.02% variability and was positively correlated to all the grain yield, quality parameters and morpho-metric parameters except phytic acid and PA/Zn (Table 3). PC2 contributed 17.94% and phytate and its ratio to Zn contributed mainly to component 2. Similarly, PC3 accounted for 15.58% variation and had a profound positive association with Zn and Fe. PC4 depicted 11.72% variability with a significant positive association with Zn, seed protein, and phytate. PCA biplot based on PC1 and PC2 accounting for 52.96% variation showed the presence of two groups under stress (Figure 2B). All the seed size, yield, and quality traits contributed for PC1 and group II genotypes had a great association with the traits referring to PC1. PA and its ratio to Zn were the major traits for PC2 and group I genotypes were strongly correlated to these traits.

### 3.6. Identification of potential genotypes by clustering

**Figure 3:**
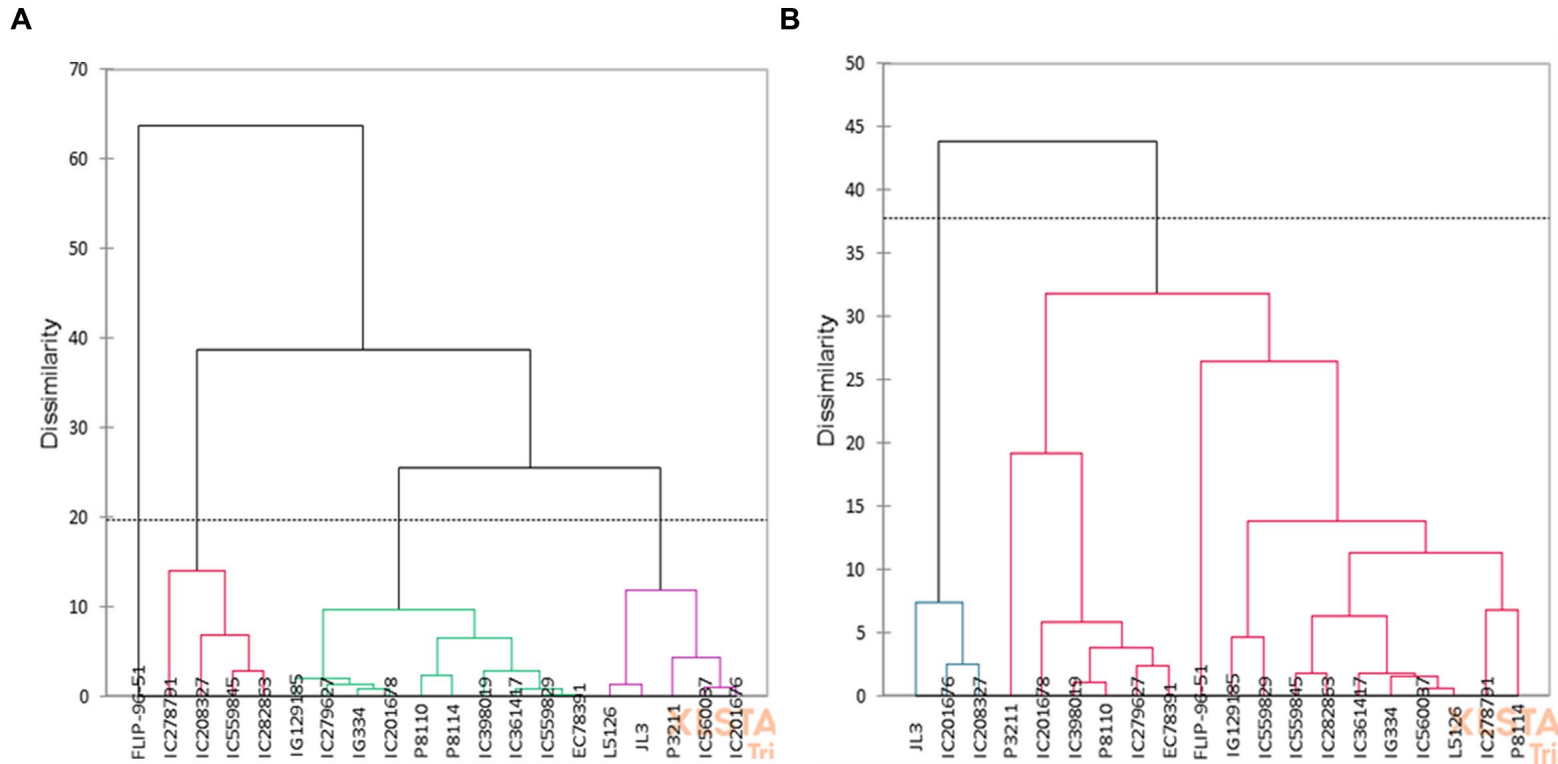
Dendrogram representing the clustering in **(A)** normal and **(B)** water stress conditions.

The hierarchical cluster analysis was performed following Ward’s method considering all the recorded traits under normal and water limited conditions. The genotypes were grouped into 4 clusters each in normal and stress environment (Figure 3). In no stress condition, the genotypes were put together into two major clusters at a distance of 38% under normal condition (Figure 3A). Cluster I had FLIP-96-51. Second cluster was further divided into 3 sub-clusters., cluster II IC278791, IC208327, IC559845 and IC282863, cluster III IG129185, C279627, IG334, IC201678, P8110, P8114, IC398019, IC361417, IC559829, EC78391 and cluster IV L5126, JL3, P3211, IC560037 and IC201676 (Figure 3A).

In stress, genotypes were classified into two major clusters (Figure 3B). Cluster I consisted of JL3, IC201676, IC208327 and Cluster 2 had the rest of the genotypes and was further sub divided into small subclusters. The most diverse genotypes, JL3 and P8114, were identified along the dendrogram’s edge. Accessions within each cluster showed less variation, however among the clusters a significant difference was observed with respect to grain yield and quality traits. Cluster 1 (JL3, IC201676, IC208327) recorded poor performance with respect to seed size, yield and quality traits and reported higher values for phytate and PA/Zn ratio under stress (Figure 2B). In cluster II, IG129185, IC559845, IC599829, IC282863, IC361417, IG334, IC560037, P8114 and L5126 were promising with respect to seed size, Fe and protein content (Figures 2B, 3B). FLIP-96-51, P3211, IC398019, and P8110 performed better in terms of yield, Zn, and DPPH radical scavenging activity (Figures 2B, 3B).

## 4. Discussion

Lentil is sensitive to water limitation during the seedling and flowering stage. Severe water stress may reduce the crop yield by 50% depending on the stage (18). In the study, the effect of water stress was studied on yield, seed size and quality traits in lentils. Stress reduced seed Fe, Zn, protein, and DPPH radical scavenging capacity in different lentil genotypes (Table 1). In contrast, there was an increase in mean length, breadth, area, phytate, and PA/Zn ratio in all the tested genotypes.

Seed yield and test weight reduced in genotypes on exposure to stress and G, E, and their interaction effects were significant at *p* < 0.001. As already reported in different studies, impairment of physiological mechanism, photosynthate mobilization, and loss of pods was associated with the yield loss in lentil under stress condition (24–26).

Genetic constitution and environmental factors affect the seed composition in legumes significantly (27). Grain mineral and protein content have a profound correlation to environmental conditions. Present reported significant reduction in Fe and Zn content in different lentil genotypes in stress. Water limitation induced decrease in grain Fe and Zn content may be due to hampered nutrient uptake, availability, transport and unloading mechanism (28, 29). In addition, nutrient absorption and utilization efficiency may further decline due to slow transpiration rate under water stress (26, 30). Though we reported low mineral content in lentil, on the contrary, water deficit had no effect on seed mineral content in common bean (31).

The PA is an important form of phosphorus storage in legumes. It is required during germination and the early stages of plant growth. It is a potential chelator of cations and can bind with minerals in the digestive tract available by food consumption. Therefore, PA poses a constraint to nutrient absorption and may lead to deficiency. Though the lentil germplasm had significant variability for Fe and Zn content, but the presence of antinutrients like PA may limit the intake of micronutrients. Therefore, in the present study PA and its ratio to Zn were quantified under normal and water stress conditions. We observed a highly significant increase in PA and a reduction in Zn availability because of stress. Dumschott et al. (32) reported the accumulation of phytic acid like myo-inositol hexa phosphoric acid during water scarcity observed in the present study as well. High PA concentration was reported in lentils and common beans under the high temperature regimes (33, 34) and also under combined heat and water stress (24, 26). Water limitation downregulates the protein synthesis. In addition, poor N fixation and partitioning also result in low protein in legumes under drought conditions (18). We also observed mean reduction of 25.3% in the protein content of the lentil genotypes (18) and chickpeas (11). However, the highest protein content was 25.81 and 22.5% under normal and stress conditions, respectively, (Table 1), which shows that response was genotype dependent. Lentil is rich in polyphenols, which have antioxidant, anti-inflammatory, and nephro-protective properties. Lentil consumption is recommended to decrease the risk of diabetes, obesity, and other cardio-vascular diseases. Mean antioxidant activity quantified using DPPH asaay declined dramatically under stress. Reduction in antioxidant capacity may be attributed to a decrease in phenol concentration as reported in cow pea (35). Correlation study showed that grain yield was positively related to antioxidant properties in stress. Though mean antioxidant activity reduced, genotypes with higher yield may had comparatively more antioxidant capacity. Seed size is indirectly determined by measuring the test weight of seeds in majority of the studies (36). But seed weight does not represent the seed size precisely. In the present study, seeds were scanned, and images were analyzed to measure seed length, breadth and area under both the conditions. We noticed an increase in seed shape parameters in stress conditions. Mean length and breadth increased slightly, while the change in seed area was the most significant (Table 1). The range for different parameters revealed that minimum seed length, breadth, and area increased slightly, while maximum reduced (Except length) in water- limited environment (Table 1). Correlation analysis showed that seed size traits were correlated to grain weight under normal condition and no correlation existed in water stress. Shrestha et al. (37) reported 40 and 30% reduction in flower formation and seed abortion, respectively, in lentil under water deficit. Since these parameters were not recorded in the present study, there is a possibility that due to high seed abortion, seed length might have increased. However, there was no significant difference between control and water stress conditions for pod related traits in the present investigation, intensity of water stress imposed in both the studies may be the reason for the difference in the trait response. Another possible reason for increase in grain size may be genotypic adaptation in response to drier conditions as observed in wild lupin in contrast to cultivated lupin species (38). In this regard, a more detailed study considering the seed coat thickness and role of pod wall may be carried out. Similar findings were documented in lentils under heat (39) and soybean (40). As hypothesized, we reported reduced seed yield along with poor quality in different lentil genotypes in response to water limitation.

Correlation analysis showed presence of positive correlation between antioxidant properties and grain yield under water stress, which may be due to tolerance in lentil genotypes for water stress. Lentil genotypes with higher Zn content and availability were able to maintain higher grain weight by maintain better plant water relations, antioxidative potential, stomatal regulation and photosynthesis in stress as reflected by its positive correlation to TW in stress. PCA analysis defined major traits contributing to total variability in the studied genotypes, which was mainly explained by yield and nutritional traits as reported in previous studies in lentils (18, 39). Clustering identified four groups under normal and two groups under stress conditions. Genotypes present in Cluster I had greater decline in seed quality traits (Fe, Zn, PRT and DPPH radical scavenging activity) compared to cluster II and its sub-clusters (Figures 2, 3). Cluster I genotype showed more susceptibility to water stress. Similar findings were previously reported in lentils (41, 42). Based on clustering, genotypes IG129185, IC559845, IC599829, IC282863, IC361417, IG334, IC560037, P8114 and L5126 were promising for seed size, Fe and protein content and FLIP-96-51, P3211, IC398019 promising for yield, Zn and antioxidant properties. By focusing on seed quality traits along with seed yield, we could identify promising genotypes, which can be target for quality breeding in drought prone environment. Identified genotypes can also be used for further detailed study of Fe/Zn uptake, transport and availability during water scarcity in lentil.

## 5. Conclusion

Our findings revealed that water stress not only reduced yield but also deteriorated grain quality in lentils, therefore breeding for water limited environment should also take grain quality into consideration. Genotypic responses were significantly different with respect to the yield and quality in different moisture regimes, which may be attributed to the diversity prevailing in the studied genotypes. Positive correlation among yield and antioxidant property, grain Zn and TW in stress may be investigated and validated further with large number of genotypes.Identified genotypes IG129185, IC559845, IC599829, IC282863, IC361417, IG334, IC560037, P8114 and L5126, FLIP-96-51, P3211, IC398019 may serve as potential donor for different yield and quality traits in lentil genetic improvement or biofortification.

## Data availability statement

The raw data supporting the conclusions of this article will be made available by the authors, without undue reservation.

## Author contributions

RB: conceptualization, resources, supervision, manuscript writing, and finalization. RSB: resources, manuscript writing, and editing. HD: resources and manuscript editing. HS and SP: investigation. SuK: data analysis and manuscript writing. NK: data analysis. MA: resources and manuscript writing. SG: manuscript editing and supervision. ShK: resources and supervision. All authors contributed to the article and approved the submitted version.

## Funding

The study was supported by the Department of Biotechnology, India (BT/PR30790/BIC/101/1185/2018) and the CGIAR Research program on grain legumes and dryland cereals.

## Conflict of interest

The authors declare that the research was conducted in the absence of any commercial or financial relationships that could be construed as a potential conflict of interest.

## Publisher’s note

All claims expressed in this article are solely those of the authors and do not necessarily represent those of their affiliated organizations, or those of the publisher, the editors and the reviewers. Any product that may be evaluated in this article, or claim that may be made by its manufacturer, is not guaranteed or endorsed by the publisher.

## References

[1] De Moura Ariza Alpino T Mazoto ML De Barros DC De Freitas CM. The impacts of climate change on food and nutritional security: a literature review. Ciên Saúde Colet. (2022) 27:273–86. doi: 10.1590/1413-81232022271.05972020, PMID: 35043907

[2] Fan S. *Food policy in 2018–2019: Growing urgency to address the SDGs*. International food policy research institute (IFPRI), 2019 global food policy report. (2019).

[3] Devirian TA Volpe SL. The physiological effects of dietary boron. Crit Rev Food Sci Nutr. (2003) 43:219–31. doi: 10.1080/10408690390826491 12705642

[4] Chen PX Bozzo GG Freixas-Coutin JA Marcone MF Pauls PK Tang Y. Free and conjugated phenolic compounds and their antioxidant activities in regular and non-darkening cranberry bean (*Phaseolus vulgaris* L.) seed coats. J Funct Foods. (2015) 18:1047–56. doi: 10.1016/j.jff.2014.10.032

[5] Qados AMA. Effect of salt stress on plant growth and metabolism of bean plant *Vicia faba* (L.). J Saudi Soc Agric Sci. (2011) 10:7–15. doi: 10.1016/j.jssas.2010.06.002

[6] Sehgal A Sita K Bhandari K Kumar S Kumar J Vara Prasad PV. Influence of drought and heat stress, applied independently or in combination during seed development, on qualitative and quantitative aspects of seeds of lentil (*Lens culinaris* Medikus) genotypes, differing in drought sensitivity. Plant Cell Environ. (2019) 42:198–211. doi: 10.1111/pce.13328, PMID: 29744880

[7] Plaut Z Butow BJ Blumenthal CS Wrigley CW. Transport of dry matter into developing wheat kernels and its contribution to grain yield under post-anthesis water deficit and elevated temperature. Field Crops Res. (2004) 86:185–98. doi: 10.1016/j.fcr.2003.08.005

[8] Farooq M Gogoi N Barthakur S Baroowa B Bharadwaj N Alghamdi SS. Drought stress in grain legumes during reproduction and grain filling. J Agron Crop Sci. (2017) 203:81–102. doi: 10.1111/jac.12169

[9] Farooq M Nadeem F Gogoi N Ullah A Alghamdi SS Nayyar H. Heat stress in grain legumes during reproductive and grain-filling phases. Crop Pasture Sci. (2017) 68:985–1005. doi: 10.1071/CP17012

[10] Rakszegi M Darko E Lovegrove A Molnár I Lang L Bedő Z. Drought stress affects the protein and dietary fiber content of wholemeal wheat flour in wheat/*Aegilops* addition lines. PLoS One. (2019) 14:e0211892. doi: 10.1371/journal.pone.0211892, PMID: 30721262 PMC6363227

[11] Ashrafi V Pourbozorg H Kor NM Ajirloo AR Shamsizadeh M Shaaban M. Study on seed protein and protein profile pattern of chickpea (*Cicer arietinum* L.) by SDS-PAGE under drought stress and fertilization. Int J Life Sci. (2015) 9:87–90. doi: 10.3126/ijls.v9i5.12704

[12] Mumtaz MZ Saqib M Abbas G Akhtar J Ul-Qamar Z. Drought stress impairs grain yield and quality of rice genotypes by impaired photosynthetic attributes and K nutrition. Rice Sci. (2020) 27:5–9. doi: 10.1016/j.rsci.2019.12.001

[13] Ardakani LG Farajee H Kelidari A. The effect of water stress on grain yield and protein of spotted bean (*Phaseolus vulgaris* L.), cultivar Talash. Int J Adv Biol Biomed Res. (2013) 1:940–9.

[14] FAOSTAT. *Statistics database*. Food and Agriculture Organization of the United Nations, Rome, Italy. (2020). Available at: https://www.fao.org/faostat.

[15] Thavarajah D Johnson CR McGee R Thavarajah P. Phenotyping nutritional and antinutritional traits. In: Phenomics of crop plants: trends, and options and limitations. New Delhi: Springer (2015) 223–233.

[16] Johnson CR Combs GF Jr Thavarajah P. Lentil (*Lens culinaris* L.): a prebiotic-rich whole food legume. Food Res Int. (2013) 51:107–13. doi: 10.1016/j.foodres.2012.11.025

[17] Baroowa B Gogoi N. Biochemical changes in black gram and green gram genotypes after imposition of drought stress. J Food Legumes. (2014) 27:350–3.

[18] Choukri H Hejjaoui K El-Baouchi A El Haddad N Smouni A Maalouf F. Heat and drought stress impact on phenology, grain yield and nutritional quality of lentil (*Lens culinaris* Medikus). Front Nutr. (2020) 7:596307. doi: 10.3389/fnut.2020.596307, PMID: 33330596 PMC7719779

[19] Pang J Turner NC Khan T Du YL Xiong JL Colmer TD. Response of chickpea (*Cicer arietinum* L.) to terminal drought: leaf stomatal conductance, pod abscisic acid concentration, and seed set. J Exp Bot. (2017) 68:1973–85. doi: 10.1093/jxb/erw153, PMID: 27099375 PMC5429003

[20] AOAC. Official methods of analysis. 15th ed. Arlington, VA: Association of Official Analytical Chemist (1990).

[21] Kjeldahl C. A new method for the determination of nitrogen in organic matter. Anal Chem. (1983) 22:366–82. doi: 10.1007/BF01338151

[22] Megazyme Phytic Acid Assay Kit. (2016). Available at: https://www.megazyme.com/phytic-acid-assay-kit.

[23] Brand-Williams W Cuvelier ME Berset C. Use of a free radical method to evaluate antioxidant activity. LWT. (1995) 28:25–30. doi: 10.1016/S0023-6438(95)80008-5

[24] Awasthi R Kaushal N Vadez V Turner NC Berger J Siddique KH. Individual and combined effects of transient drought and heat stress on carbon assimilation and seed filling in chickpea. Funct Plant Biol. (2014) 41:1148–67. doi: 10.1071/fp13340, PMID: 32481065

[25] Delahunty A Nuttall J Nicolas M Brand J. Response of lentil to high temperature under variable water supply and carbon dioxide enrichment. Crop Pasture Sci. (2018) 69:1103–12. doi: 10.1071/CP18004

[26] Sehgal A Sita K Kumar J Kumar S Singh S Siddique KHM. Effects of drought, heat and their interaction on the growth, yield and photosynthetic function of lentil (*Lens culinaris* Medikus) genotypes varying in heat and drought sensitivity. Front Plant Sci. (2017) 8:1776. doi: 10.3389/fpls.2017.01776, PMID: 29089954 PMC5651046

[27] Grusak MA. Enhancing mineral content in plant food products. J Am Coll Nutr. (2002) 21:178S–83S. doi: 10.1080/07315724.2002.10719263 12071302

[28] Huang B Rachmilevitch S Xu J. Root carbon and protein metabolism associated with heat tolerance. J Exp Bot. (2012) 63:3455–65. doi: 10.1093/jxb/ers003, PMID: 22328905

[29] Sánchez-Rodríguez E del Mar Rubio-Wilhelmi M Cervilla LM Blasco B Rios JJ Leyva R. Study of the ionome and uptake fluxes in cherry tomato plants under moderate water stress conditions. Plant Soil. (2010) 335:339–47. doi: 10.1007/s11104-010-0422-2

[30] Bista DR Heckathorn SA Jayawardena DM Mishra S Boldt JK. Effects of drought on nutrient uptake and the levels of nutrient-uptake proteins in roots of drought-sensitive and-tolerant grasses. Plan Theory. (2018) 7:28. doi: 10.3390/plants7020028, PMID: 29601475 PMC6027393

[31] Smith MR Veneklaas E Polania J Rao IM Beebe SE Merchant A. Field drought conditions impact yield but not nutritional quality of the seed in common bean (*Phaseolus vulgaris* L.). PLoS One. (2019) 14:e0217099. doi: 10.1371/journal.pone.0217099, PMID: 31170187 PMC6553706

[32] Dumschott K Richter A Loescher W Merchant A. Post photosynthetic carbon partitioning to sugar alcohols and consequences for plant growth. Phytochemistry. (2017) 144:243–52. doi: 10.1016/j.phytochem.2017.09.019, PMID: 28985572

[33] Hummel M Hallahan BF Brychkova G Ramirez-Villegas J Guwela V Chataika B. Reduction in nutritional quality and growing area suitability of common bean under climate change induced drought stress in Africa. Sci Rep. (2018) 8:1–11. doi: 10.1038/s41598-018-33952-4 30385766 PMC6212502

[34] Thavarajah P See CT Vandenberg A. Phytic acid and Fe and Zn concentration in lentil (*Lens culinaris* L.) seeds is influenced by temperature during seed filling period. Food Chem. (2010) 122:254–9. doi: 10.1016/j.foodchem.2010.02.073

[35] Carvalho M Gouvinhas I Castro I Matos M Rosa E Carnide V. Drought stress effect on polyphenolic content and antioxidant capacity of cowpea pods and seeds. J Agron Crop Sci. (2021) 207:197–207. doi: 10.1111/jac.12454

[36] Tullu A Kusmenoglu I McPhee KE Muehlbauer FJ. Characterization of core collection of lentil germplasm for phenology, morphology, seed and straw yields. Genet Resour Crop Evol. (2001) 48:143–52. doi: 10.1023/A:1011254629628

[37] Shrestha R Turner NC Siddique KHM Turner DW. Physiological and seed yield responses to water deficits among lentil genotypes from diverse origins. Aust J Agric Res. (2006) 57:903–15. doi: 10.1071/AR05204

[38] Berger JD Shrestha D Ludwig C. Reproductive strategies in mediterranean legumes: trade-offs between phenology, seed size and vigor within and between wild and domesticated *Lupinus* species collected along aridity gradients. Front Plant Sci. (2017) 8:548. doi: 10.3389/fpls.2017.00548, PMID: 28450875 PMC5390039

[39] Choukri H El Haddad N Aloui K Hejjaoui K El-Baouchi A Smouni A. Effect of high temperature stress during the reproductive stage on grain yield and nutritional quality of lentil (*Lens culinaris* Medikus). Front Nutr. (2022) 9:857469. doi: 10.3389/fnut.2022.857469, PMID: 35495922 PMC9051399

[40] Wang X Fu G Yuan S Zhang H. Genetic diversity analysis of seed appearance quality of Chinese soybean mini core collection. Proc Eng. (2011) 18:392–7. doi: 10.1016/j.proeng.2011.11.063

[41] Kumar H Dikshit HK Singh A Jain N Kumari J Singh AM. Characterization of grain iron and zinc in lentil ('*Lens culinaris*' Medikus' culinaris') and analysis of their genetic diversity using SSR markers. Aust J Crop Sci. (2014) 8:1005–12.

[42] Thavarajah D Thavarajah P Sarker A Vandenberg A. Lentils (*Lens culinaris* Medikus subspecies culinaris): a whole food for increased iron and zinc intake. J Agric Food Chem. (2009) 57:5413–9. doi: 10.1021/jf900786e, PMID: 19459707

